# Morphological profile and physical activity readiness among Portuguese police officers in the Lisbon metropolitan area

**DOI:** 10.47626/1679-4435-2026-1544

**Published:** 2026-07-26

**Authors:** Inês Sousa, Catarina N. Matias, Sérgio Ramos, Vanessa Santos, Luís Miguel Massuça

**Affiliations:** 1 ISCPSI - Instituto Superior de Ciências Policiais e Segurança Interna, Lisboa, Portugal; 2 CIDEFES - Centro de Investigação em Desporto, Educação Física, Exercício e Saúde, Universidade Lusófona e CIFI2D - Centro de Investigação da Faculdade de Desporto da Universidade do Porto, Universidade do Porto, Lisboa e Porto, Portugal; 3 Piaget Research Center for Ecological Human Development, Instituto Piaget, Almada, Lisboa, Portugal; 4 CIPER - Exercise and Health Laboratory, Centro Interdisciplinar de Performance Humana, Faculdade de Motricidade Humana, Universidade de Lisboa, Cruz-Quebrada, Lisboa, Portugal; 5 ICPOL - Centro de Investigação Policial, Instituto Superior de Ciências Policiais e Segurança Interna, Lisboa, Portugal

**Keywords:** age groups, body composition, exercise, health of specific groups, work capacity evaluation

## Abstract

**Introduction:**

Maintaining body fat levels within recommended ranges is essential for optimal physical performance among police officers.

**Objectives:**

To determine the morphological profile and readiness for physical activity among police officers according to sex and age group.

**Methods:**

This cross-sectional study included 546 police officers assigned to the Lisbon metropolitan area. Morphological assessment was performed using anthropometric measurements and bioelectrical impedance analysis. Readiness for physical activity was assessed using the Physical Activity Readiness Questionnaire.

**Results:**

Among the participants, 38.5% had a normal body mass index, 78.2% had a waist circumference below the recommended cutoff value, 67.8% had a normal conicity index, 59.9% had a metabolic age equal to or lower than their chronological age, 89.6% presented healthy total body water levels, 52.9% had a healthy body fat percentage, and 65.8% were classified as ready for physical activity. Conversely, 49.6% of officers were classified as overweight and 11.6% as obese according to body mass index. High or very high waist circumference was observed in 14.7% of participants, while 32.2% presented an increased conicity index. A metabolic age greater than chronological age was identified in 40.1% of officers. Total body water levels were below the healthy range in 8.4% and above the healthy range in 2.0% of participants. Regarding body fat percentage, 30.0% were classified as overweight and 13.7% as obese. Restrictions to physical activity readiness were identified in 21.2% of officers, while 13.0% were considered completely unfit for physical activity.

**Conclusions:**

Most police officers demonstrated an acceptable morphological profile. However, the high prevalence of excess body weight and adiposity indicates potential health and fitness concerns, which are reflected in limitations regarding readiness for physical activity. These findings highlight the importance of regular monitoring and targeted interventions aimed at weight management and the promotion of physical fitness within police forces.

## INTRODUCTION

Police forces play a fundamental role in safeguarding the public interest and protecting citizens’ legally protected rights, with the primary objective of upholding democratic legality, ensuring internal security, and guaranteeing citizens’ rights. Police work is inherently broad and complex, encompassing a wide range of situations, including traffic accident investigations, suspect pursuits, and the maintenance of public order [^1^]. Despite the high cognitive demands and responsibilities associated with police duties, police work is often characterized by prolonged periods of sedentary behavior [^1^], interspersed with sudden episodes of intense physical exertion that may lead police officers (POs) to physical exhaustion, such as arrests involving resistance to law enforcement, shootings, robberies, and traffic accidents [^2^,^3^]. To perform their duties effectively and respond to the unpredictability of their mission while minimizing operational risks, POs require above-average levels of physical fitness (PF) [^4^]. However, these requirements are adversely affected by the high prevalence of sedentary behavior associated with the profession [^5^].

Although police recruitment processes typically include mandatory PF assessments [^6^], subsequent professional assignments, particularly among lower-ranking POs, often involve predominantly administrative tasks. Combined with factors such as limited organizational support for physical activity (PA), technological advances, self-policing behaviors, and presenteeism [^1^], these conditions may contribute to declines in PF and increases in adiposity [^7^], thereby exacerbating obesity rates among police personnel [^8^]. This scenario is further compounded by shift work and demanding occupational routines [^9^], which expose POs to increased levels of stress and sleepiness [^5^], as well as unhealthy dietary habits that favor fat accumulation and reductions in muscle mass (MM) [^9^].

In this context, previous studies suggest that police competence and operational performance are positively influenced by PF, including both physical capacity and body composition [^10^]. It is well established that the levels of PA during young adulthood predict PF in middle age. Beyond this stage, occupational performance tends to decline more markedly [^2^]. Furthermore, the literature indicates that PF gradually decreases throughout the aging process, with a more pronounced decline after the age of 50, at an estimated rate of approximately 1% per year [^11^]. Therefore, monitoring not only PF but also morphological characteristics and readiness for occupational demands is essential among POs.

In a longitudinal study, Sörensen et al. [^2^] demonstrated a positive association between sports participation and PF over a 15-year period, concluding that police organizations should implement incentives and requirements that promote active and healthy lifestyles from the beginning of POs’ careers. Greater investment in physical education during youth contributes to better PF and health outcomes later in life and may help prevent the development of noncommunicable diseases, including cardiovascular disease, chronic respiratory diseases, diabetes, and obesity [^5^].

Similarly, Demling & DeSanti [^9^] reported that POs tend to gain weight (Wt) and progressively lose strength after completing their initial training. According to the authors, the primary contributors to these changes were shift work, poor dietary habits, and the lack of structured training programs. These occupational factors negatively affect PF, job performance, and overall health by increasing the risk of injuries and several chronic conditions, including obesity and metabolic syndrome, both of which occur more frequently among POs than in the general population [^5^]. Conversely, adherence to a healthy lifestyle has been associated with improved police performance, thereby contributing to greater public safety and community well-being [^12^]. Likewise, maintaining fat mass levels within healthy ranges, combined with higher PF levels, has been shown to improve overall health and increase the likelihood of optimal occupational performance among POs [^13^].

Despite the growing body of research on police health and PF, studies describing the morphological characteristics of personnel from the Portuguese Public Security Police (PSP) remain scarce. Therefore, the present study aimed to determine the morphological profile and PA readiness among POs assigned to the Lisbon metropolitan area, according to sex and age group. We hypothesized that anthropometric and body composition parameters would be significantly associated with levels of PA readiness among POs.

## METHODS

### STUDY DESIGN

This cross-sectional study was conducted in three stages: i) collection of sociodemographic and occupational data, ii) morphological assessment, and iii) assessment of PA habits. The study was conducted in accordance with the principles of the Declaration of Helsinki and received approval from the Instituto Superior de Ciências Policiais e Segurança Interna (ISCPSI) and the PSP (reference no. 12/SECDE/2024, January 25, 2024).

### PARTICIPANTS

The study was conducted among POs serving in the Lisbon metropolitan area of the PSP, specifically those assigned to the National Directorate (ND), the ISCPSI, and the Lisbon Metropolitan Command (COMETLIS). All participants provided written informed consent prior to participation, and data collection took place between January and April 2024.

A total of 546 POs participated in the study, including 29 from ISCPSI, 48 from the ND, and 469 from COMETLIS. The latter group comprised POs from the Sintra Police Division (n = 23), Oeiras Police Division (n = 25), 3rd Police Division (n = 25), Loures Police Division (n = 27), Amadora Police Division (n = 30), Vila Franca de Xira Police Division (n = 45), 4th Police Division (n = 46), 2nd Police Division (n = 47), the headquarters of the Lisbon Metropolitan Command (n = 54), and Cascais Police Division (n = 147).

The sample was statistically representative of the target population, with a 99% confidence level and a 5% margin of error. Table 1 presents a detailed characterization of participants.

**Table 1.** Distribution of participants according to sex and age group

Variables		Female (n = 90)	Male (n = 456)
Age group, years	≤ 29	16	103
	30-39	22	138
	40-49	20	109
	≥ 50	32	106
Professional category	Police officer	69	386
	Chief of police	15	47
	Senior police officer	6	23

### INSTRUMENTS AND PROCEDURES

After collecting participants’ sociodemographic and occupational characteristics, including i) sex (female or male), ii) age (years), and iii) professional category (i.e., police officer, chief of police, or senior police officer), morphological assessments and PA readiness evaluations were conducted at police facilities in a private, comfortable setting.

To ensure procedural standardization, participants were instructed to comply with the recommendations proposed by Heyward [^14^], preferably undergoing assessment in the morning. Specifically, participants were asked to refrain from using diuretic medications during the previous 7 days, consuming alcohol within the previous 48 hours, eating or drinking within the hours preceding the assessment, engaging in moderate-to-vigorous PA during the previous 12 hours, and urinating within 30 minutes before the assessment.

#### Morphological assessment

The morphological assessment included the following variables: i) height (Ht), ii) Wt, iii) body mass index (BMI), iv) hip circumference (HC), v) waist circumference (WC), vi) waist-to-height ratio (WHtR), vii) waist-to-hip ratio (WHR), and viii) conicity index (CI).

Ht was measured using a wall-mounted stadiometer (Holtain Ltd., Crymych, UK) with a precision of 0.1 cm. Participants stood barefoot in an upright position, with their heels together, arms relaxed alongside the body, Wt evenly distributed between both feet, and head positioned in the Frankfurt plane following a normal exhalation [^15^].

Wt was measured using a single-frequency bioelectrical impedance analyzer (Tanita InnerScan V BC-601 Gold, Tanita Ltd., Netherlands) positioned on a stable and level surface, according to the manufacturer’s instructions [^15^].

BMI was calculated as Wt (kg) divided by Ht squared (m^2^) (Equation 1). Participants were classified as underweight (< 18.5 kg/m^2^), normal Wt (18.5-24.9 kg/m^2^), overweight (25.0-29.9 kg/m^2^), obesity class I (30.0-34.9 kg/m^2^), or obesity class II (35.0-39.9 kg/m^2^) [^16^].


Equation 1 $$\text{IMC}=\frac{\text{PC}\left(\text{kg}\right)}{\text{Est}{\left(\text{m}\right)}^{2}}$$

HC was measured at the point of greatest gluteal protuberance using a Harpenden anthropometric tape (Holtain Ltd., Crymych, UK), following World Health Organization guidelines [^15^]. The tape was placed around the participant without compressing the skin.

WC was measured at the midpoint between the lowest rib and the iliac crest [^17^]. Measurements were obtained with the participant standing upright, abdomen relaxed, arms hanging naturally at the sides, feet together, and Wt evenly distributed. The tape was positioned horizontally around the abdomen without compressing the skin and measurements were recorded after normal expiration [^15^]. WC values were classified as follows: i) very low, < 70 cm for females and < 80 cm for males; ii) low, 70-89 cm for females and 80-99 cm for males; iii) high, 90-110 cm for females and 100-120 cm for males; and iv) very high, > 110 cm for females and > 120 cm for males [^18^].

WHR and WHtR were calculated using Equations 2 and 3, respectively.


Equation 2 $$\text{RCQ}=\frac{\text{CC}\left(\text{cm}\right)}{\text{CQ}\left(\text{cm}\right)}$$

Equation 3 $$\text{RCEst}=\frac{\text{CC}\left(\text{cm}\right)}{\text{Est}\left(\text{cm}\right)}$$

CI was calculated according to Equation 4 and classified as normal (< 1.18 for females and < 1.25 for males) or increased (≥ 1.18 for females and ≥ 1.25 for males) [^19^].


Equation 4 $$\text{IC}=\frac{\text{CC}\left(\text{m}\right)}{0,109\sqrt{\frac{\text{PC}\left(\text{kg}\right)}{\text{Est}\left(\text{m}\right)}}}$$

#### Assessment of body composition

Body composition was assessed using a single-frequency bioelectrical impedance analyzer (Tanita InnerScan V BC-601 Gold; Tanita Ltd., Netherlands). Participants were instructed to remove their socks and any metallic objects before the assessment, which was performed with participants standing upright. Proper contact was ensured between the electrodes and the fingers and soles of the feet, with the arms extended away from the trunk and the legs slightly apart.

Through direct contact with the four electrodes [^20^], the device generates a low-frequency electrical current (50 kHz) that primarily passes through extracellular fluids, allowing the measurement of tissue resistance, as the current does not cross cell membranes [^19^]. The following variables were obtained: i) metabolic age (MA); ii) total body water (TBW); iii) bone mass (BM); iv) fat mass percentage (%FM); and v) MM.

MA was classified as either ≤ chronological age (CA) or > CA. TBW was classified as below healthy (< 45% for females and < 50% for males), healthy (45%-60% for females and 50%-65% for males), or above healthy (> 60% for females and > 65% for males) [^21^]. %FM was classified as underweight, healthy, overweight, or obese according to established reference values [^22^].

#### PA readiness

PA readiness was assessed using the Physical Activity Readiness Questionnaire (PAR-Q) [^23^], which consists of seven dichotomous questions (yes/no) designed to identify signs, symptoms, or diagnoses of cardiovascular, pulmonary, or metabolic disease and to determine whether an individual can safely engage in PA.

PAR-Q results were classified as follows: i) able to perform PA (0 affirmative responses); ii) able to perform PA with restrictions (one affirmative response); and iii) unable to perform PA and requiring medical evaluation (≥ two affirmative responses).

### STATISTICAL ANALYSIS

Descriptive statistics were used to characterize the sample and are presented as mean ± SD.

Associations between categorical variables were assessed using the chi-square test of independence. Specifically, the analyses examined whether BMI classification, WC classification, CI classification, MA status, TBW classification, %FM classification, and PA readiness differed according to sex (female or male) and age group (≤ 29 years, 30-39 years, 40-49 years, and ≥ 50 years).

Because some variables did not meet the assumptions required for parametric analyses, nonparametric tests were employed. Differences between female and male POs in anthropometric and body composition variables were evaluated using the Mann-Whitney *U* test. The variables analyzed included Ht, Wt, HC, WC, BMI, WHR, WHtR, CI, MA, TBW, BM, %FM, and MM.

The Kruskal-Wallis test, followed by post hoc multiple comparisons, was used to assess differences across age groups within each sex for the same set of morphological and body composition variables.

Statistical significance was set at p < 0.05 for all analyses. Data were analyzed using IBM SPSS Statistics for Windows, version 28.0 (IBM Corp., Armonk, N.Y., USA).

## RESULTS

Table 2 shows the morphological characteristics of the POs.

**Table 2.** Morphological characteristics of the overall sample and according to sex

Variables	Total		Female (n = 90)			Male (n = 456)			Mann-Whitney *U* test		
	Mean	SD	Mean	SD	Rank	Mean	SD	Rank	*U*	W	p-value
Age, years	40.20	11.20	42.70	11.80	307.90	39.70	11.00	266.70	17426.50	121622.50	*
Ht, cm	174.90	7.30	165.60	4.30	77.00	176.70	6.40	312.30	38207.50	142403.50	***
Wt, kg	80.00	12.50	64.90	7.30	81.30	82.90	11.10	311.40	37822.00	142018.00	***
BMI, kg/m^2^	26.10	3.20	23.70	2.80	156.00	26.50	3.10	296.70	31098.00	135294.00	***
HC, cm	102.40	6.00	99.30	5.70	190.80	103.10	5.90	289.80	27964.50	132160.50	***
WC, cm	89.60	9.80	78.80	8.80	110.20	91.70	8.50	305.70	35213.50	139409.50	***
WHR	0.87	0.07	0.79	0.06	102.10	0.89	0.06	307.30	35950.00	140146.00	***
WHtR	0.51	0.05	0.48	0.06	169.70	0.52	0.05	204.00	29865.50	134061.50	***
CI	1.22	0.07	1.15	0.08	153.90	1.23	0.07	297.10	31284.00	135480.00	***
MA, years	37.90	15.90	38.00	15.20	275.10	37.80	16.00	273.20	20378.50	124574.50	NS
MA vs CA, years	−2.30	10.50	−4.70	10.20	237.90	−1.90	10.50	280.50	23724.50	127920.50	*
TBW, %	55.60	5.00	51.30	4.90	142.60	56.40	4.50	299.30	32300.00	136496.00	***
BM, kg	3.10	0.50	2.30	0.20	46.80	3.30	0.30	318.30	40926.50	145122.50	***
%FM	22.40	6.90	30.30	6.30	438.50	20.80	5.90	240.90	5670.00	109866.00	***
MM, kg	58.90	9.50	42.70	3.40	47.10	62.00	6.60	318.20	40893.50	145089.50	***

BM = bone mass; BMI = body mass index; CA = chronological age; CI = conicity index; HC = hip circumference; Ht = height; MA = metabolic age; MM = muscle mass; NS = not significant; TBW = total body water; *U* = Mann-Whitney *U* statistic; W = Wilcoxon test statistic; WC = waist circumference; WHR = waist-to-hip ratio; WHtR = waist-to-height ratio; Wt = weight; %FM = fat mass percentage.

* p < 0.05;

*** p < 0.001.

In the overall sample, 38.5% of POs had a normal BMI, 78.2% had a low WC, 67.8% had a normal CI, 59.9% had a MA lower than or equal to their CA, 89.6% had healthy TBW levels, 52.9% had a healthy %FM, and 65.8% were classified as able to perform PA.

Regarding unfavorable classifications, 49.6% of POs were classified as overweight, 10.3% as obesity class I, and 1.3% as obesity class II according to BMI. High and very high WC values were observed in 14.3% and 0.4% of participants, respectively, while 32.2% presented an increased CI. A MA greater than CA was identified in 40.1% of the sample. TBW levels were below and above the healthy range in 8.4% and 2.0% of participants, respectively. %FM was classified as overweight in 30.0% of participants and as obese in 13.7%. In addition, 21.2% of POs were classified as able to perform PA with restrictions, whereas 13.0% were classified as unable to perform PA and requiring medical evaluation.

Female POs presented significantly lower values for Ht (p < 0.001), Wt (p < 0.001), BMI (p < 0.001), HC (p < 0.001), WC (p < 0.001), WHR (p < 0.001), WHtR (p < 0.001), CI (p < 0.001), MA difference (p = 0.019), TBW (p < 0.001), BM (p < 0.001), and MM (p < 0.001) than male POs. Conversely, female POs showed significantly higher age (p = 0.024) and %FM (p < 0.001) values than their male counterparts (Table 2).

Chi-square analyses indicated that BMI classification, %FM classification, and PA readiness were significantly associated with sex (Table 3).

**Table 3.** Distribution of BMI, WC, CI, MA status, TBW, %FM, and PA readiness according to sex

Variables		Total, %	Female (n = 90), %	Male (n = 456), %	Chi-square test
BMI	Underweight	0.4	2.2	0.0	X^2^(4) = 65.250 p < 0.001
	Normal weight	38.5	72.2	31.8	
	Overweight	49.6	23.3	54.8	
	Obesity class I	10.3	2.2	11.8	
	Obesity class II	1.3	0.0	1.5	
WC	Very low	7.1	13.3	5.9	X^2^(3) = 7.033 p = 0.071
	Low	78.2	75.6	78.7	
	High	14.3	11.1	14.9	
	Very high	0.4	0.0	0.4	
CI	Normal	67.8	63.3	68.6	X^2^(1) = 0.969 p = 0.325
	Increased	32.2	36.7	31.4	
MA status	≤ CA	59.9	64.4	59.0	X^2^(1) = 0.930 p = 0.335
	> CA	40.1	35.6	41.0	
TBW	Below healthy	8.4	10.0	8.1	X^2^(2) = 0.379 p = 0.828
	Healthy	89.6	87.8	89.9	
	Above healthy	2.0	2.2	2.0	
%FM	Underweight	3.3	10.0	2.0	X^2^(3) = 22.845 p < 0.001
	Healthy	52.9	62.2	51.1	
	Overweight	30.0	20.0	32.0	
	Obese	13.7	7.8	14.9	
PA readiness	Able to perform PA	65.8	55.6	67.8	X^2^(2) = 8.795 p = 0.012
	Able to perform PA with restrictions	21.2	22.2	21.1	
	Unable to perform PA	13.0	22.2	11.2	

BMI = body mass index; CA = chronological age; CI = conicity index; MA = metabolic age; PA = physical activity; TBW = total body water; WC = waist circumference; %FM = fat mass percentage.

Among female POs, age group had a significant effect on Ht (p = 0.027), BMI (p < 0.001), WC (p < 0.001), WHR (p < 0.001), WHtR (p < 0.001), CI (p = 0.003), MA (p < 0.001), TBW (p < 0.001), and %FM (total: p < 0.001; right upper limb: p = 0.020; left upper limb: p = 0.017; right lower limb: p < 0.001; left lower limb: p < 0.001; trunk: p < 0.001).

Post hoc comparisons indicated that the highest values for most significant variables were observed in the ≥ 50-year age group. The exceptions were Ht, which was highest among POs aged 30-39 years, and TBW, which was highest among those aged ≤ 29 years (Table 4).

**Table 4.** Morphological characteristics of female POs according to age group (≤ 29 years, 30-39 years, 40-49 years, and ≥ 50 years)

Variables	≤ 29 years (1)			30-39 years (2)			40-49 years (3)			≥ 50 years (4)			Statistics			Post hoc comparisons				
	Mean	SD	Rank	Mean	SD	Rank	Mean	SD	Rank	Mean	SD	Rank	KW	df	p-value	1-3	1-4	2-3	2-4	3-4
Age, years	26.00	1.83	8.50	33.90	2.20	27.50	44.80	2.80	48.50	55.80	2.80	74.50	82.405	3	***	***	***		***	**
Ht, cm	167.40	6.00	52.00	166.70	4.30	53.10	166.00	3.60	49.55	163.80	3.10	34.50	9.151	3	*	—	—	—	—	—
Wt, kg	62.10	4.49	37.70	62.40	5.70	37.30	64.70	6.30	46.88	68.10	8.80	54.20	7.220	3	NS	—	—	—	—	—
BMI, kg/m^2^	22.30	2.35	33.60	22.50	1.90	33.60	23.50	2.50	45.50	25.40	3.00	59.70	17.385	3	***	—	**	—	**	—
HC, cm	98.10	4.01	41.40	97.30	4.80	36.90	99.30	5.60	45.65	101.30	6.60	53.40	5.753	3	NS	—	—	—	—	—
WC, cm	74.00	7.01	31.40	75.10	5.50	35.60	78.00	7.60	44.20	84.20	9.40	60.20	18.080	3	***	—	**	—	**	—
WHR	0.75	0.06	28.40	0.77	0.06	38.80	0.78	0.05	43.10	0.83	0.06	60.20	18.558	3	***	—	***	—	*	—
WHtR	0.44	0.05	30.70	0.45	0.04	33.70	0.47	0.05	43.35	0.51	0.06	62.40	23.136	3	***	—	***	—	***	—
CI	1.11	0.08	33.40	1.13	0.08	37.70	1.15	0.08	42.20	1.20	0.07	59.00	14.206	3	*	—	**	—	**	—
MA, years	23.60	8.60	19.20	28.30	9.40	27.70	39.40	9.60	50.20	51.00	12.80	68.00	50.904	3	***	**	***	*	***	—
MA vs CA, years	−2.40	8.90	53.60	−5.60	9.70	44.60	−5.40	9.60	43.73	−4.90	11.80	43.20	1.917	3	NS	—	—	—	—	—
TBW, %	54.90	5.20	62.10	53.70	3.40	60.00	50.90	3.20	43.63	48.00	4.40	28.40	26.951	3	***	—	***	—	***	—
BM, kg	2.30	0.20	47.00	2.30	0.10	49.40	2.30	0.20	44.03	2.30	0.20	43.00	0.952	3	NS	—	—	—	—	—
%FM	26.60	5.70	32.30	27.20	4.90	31.70	30.40	4.70	46.55	34.20	6.30	61.00	21.569	3	***	—	**	—	***	—
MM, kg	43.40	3.50	49.20	43.00	2.90	49.10	42.60	4.00	41.75	42.20	3.20	43.50	1.337	3	NS	—	—	—	—	—

BMI = body mass index; BM = bone mass; CA = chronological age; CI = conicity index; HC = hip circumference; Ht = height; KW = Kruskal-Wallis statistic; MA = metabolic age; MM = muscle mass; NS = not significant; PO = police officer; TBW = total body water; WC = waist circumference; WHR = waist-to-hip ratio; WHtR = waist-to-height ratio; Wt = weight; %FM = fat mass percentage.

* p < 0.05;

** p < 0.01;

*** p < 0.001.

Chi-square analyses indicated that BMI classification, WC classification, CI classification, TBW classification, %FM classification, and PA readiness were significantly associated with age group among female POs (Table 5).

**Table 5.** Distribution of BMI, WC, CI, MA status, TBW, %FM, and PA readiness among female POs according to age group (≤ 29 years, 30-39 years, 40-49 years, and ≥ 50 years)

Variables		Age group, years				Chi-square test
		≤ 29	30-39	40-49	≥ 50	
BMI	Underweight	6.3	0.0	5.0	0.0	X^2^(9) = 22.120 p = 0.009
	Normal weight	87.5	95.5	70.0	50.0	
	Overweight	6.3	4.5	25.0	43.8	
	Obesity class I	0.0	0.0	0.0	6.3	
	Obesity class II	—	—	—	—	
WC	Very low	31.3	18.2	10.0	3.1	X^2^(6) = 17.030 p = 0.009
	Low	68.8	81.8	80.0	71.9	
	High	0.0	0.0	10.0	25.0	
	Very high	0.0	0.0	0.0	0.0	
CI	Normal	81.3	72.7	70.0	43.8	X^2^(3) = 8.715 p = 0.033
	Increased	18.8	27.3	30.0	56.3	
MA status	≤ CA	56.3	68.2	70.0	62.5	X^2^(3) = 0.925 p = 0.819
	> CA	43.8	31.8	30.0	37.5	
TBW	Below healthy	0.0	0.0	5.0	25.0	X^2^(6) = 21.839 p = 0.001
	Healthy	87.5	100	95.0	75.0	
	Above healthy	12.5	0.0	0.0	0.0	
%FM	Underweight	18.8	9.1	10.0	6.3	X^2^(9) = 18.626 p = 0.029
	Healthy	75.0	72.7	65.0	46.9	
	Overweight	6.3	18.2	25.0	25.0	
	Obese	0.0	0.0	0.0	21.9	
PA readiness	Able to perform PA	68.8	59.1	65.0	40.6	X^2^(6) = 16.731 p = 0.010
	Able to perform PA with restrictions	31.3	31.8	15.0	15.6	
	Unable to perform PA	0.0	9.1	20.0	43.8	

BMI = body mass index; CA = chronological age; CI = conicity index; MA = metabolic age; PA = physical activity; Pos = police officers; TBW = total body water; WC = waist circumference; %FM = fat mass percentage.

Among male POs, age group had a significant effect on Ht (p = 0.014), Wt (p = 0.001), BMI (p < 0.001), WC (p < 0.001), WHR (p < 0.001), WHtR (p < 0.001), CI (p < 0.001), MA (p < 0.001), TBW (p < 0.001), BM (p = 0.029), %FM (all measurements, pp < 0.001), and MM (total: p = 0.018; right upper limb: p = 0.002; left upper limb: p < 0.001; right lower limb: p < 0.001; left lower limb: p < 0.001).

Post hoc comparisons indicated that the highest values for most significant variables were observed in the ≥ 50-year age group. Exceptions included Ht, BM, and total and upper-limb MM, which were highest among POs aged 30-39 years, as well as TBW and lower-limb MM, which were highest among POs aged ≤ 29 years. The results are presented in Table 6.

**Table 6.** Morphological characteristics of male POs according to age group (≤ 29 years, 30-39 years, 40-49 years, and ≥ 50 years)

Variables	≤ 29 years (1)			30-39 years (2)			40-49 years (3)			≥ 50 years (4)			Statistics			Post hoc comparisons					
	Mean	SD	Rank	Mean	SD	Rank	Mean	SD	Rank	Mean	SD	Rank	KW	df	p-value	1-2	1-3	1-4	2-3	2-4	3-4
Age, years	26.3	2.17	52.0	33.9	3.10	172.5	44.7	3.00	296.0	55.1	3.30	403.5	425.638	3	***	***	***	***	***	***	***
Ht, cm	177.4	5.90	245.7	177.8	7.10	247.5	176.0	6.00	215.7	175.4	5.90	200.2	10.582	3	*					*	
Wt, kg	80.0	9.50	192.1	82.2	11.60	220.3	83.4	9.60	242.1	86.3	12.70	260.7	15.897	3	**		*	**			
BMI, kg/m^2^	25.4	2.70	178.4	26.0	2.90	204.5	26.9	2.60	253.4	28.0	3.60	282.9	41.436	3	***		***	***	*	***	
HC, cm	102.4	5.50	214.0	102.6	6.30	218.6	103.4	5.20	241.3	103.9	6.40	242.2	4.214	3	NS						
WC, cm	87.0	6.90	151.8	89.5	6.80	195.7	92.6	6.70	253.6	98.1	9.50	320.9	98.566	3	***		***	***	**	***	***
WHR	0.85	0.04	131.1	0.87	0.04	186.99	0.90	0.04	254.3	0.94	0.05	350.7	165.313	3	***	**	***	***	***	***	***
WHtR	0.49	0.04	148.9	0.50	0.04	185.85	0.53	0.04	260.5	0.56	0.05	328.5	119.420	3	***		***	***	***	***	***
CI	1.19	0.06	149.7	1.21	0.05	188.45	1.24	0.05	247.2	1.29	0.06	338.0	124.970	3	***		***	***	**	***	***
MA, years	24.2	9.60	112.8	30.4	11.10	168.9	43.3	11.20	281.5	55.2	11.90	364.1	237.604	3	***	**	***	***	***	***	***
MA vs CA, years	−2.1	9.20	228.4	−3.5	10.40	207.3	−1.5	10.60	233.9	0.1	11.40	250.6	6.772	3	NS						
TBW, %	58.5	3.90	296.3	57.8	4.30	271.8	55.6	3.60	200.9	53.3	4.50	134.6	100.698	3	***		***	***	***	***	**
BM, kg	3.3	0.30	236.1	3.3	0.40	250.3	3.2	0.30	219.7	3.2	0.30	201.9	9.020	3	*					*	
%FM	17.6	4.80	154.3	18.7	5.40	182.0	22.0	4.60	260.5	25.4	5.70	328.3	117.027	3	***		***	***	***	***	**
MM, kg	62.4	5.90	235.8	63.1	7.10	251.3	61.7	6.50	221.3	60.7	6.30	199.1	10.050	3	*					*	

BMI = body mass index; BM = bone mass; CA = chronological age; CI = conicity index; HC = hip circumference; Ht = height; KW = Kruskal-Wallis statistic; MA = metabolic age; MM = muscle mass; NS = not significant; PO = police officer; TBW = total body water; WC = waist circumference; WHR = waist-to-hip ratio; WHtR = waist-to-height ratio; Wt = weight; %FM = fat mass percentage.

* p < 0.05;

** p < 0.01;

*** p < 0.001.

Chi-square analyses indicated that BMI classification, WC classification, CI classification, TBW classification, %FM classification, and PA readiness were significantly associated with age group among male POs (Table 7).

**Table 7.** Distribution of BMI, WC, CI, MA status, TBW, %FM, and PA readiness among male POs according to age group (≤ 29 years, 30-39 years, 40-49 years, and ≥ 50 years)

Variables		Age groups, years				Chi-square test
		≤ 29	30-39	40-49	≥ 50	
BMI	Underweight	0.0	0.0	0.0	0.0	X^2^(9) = 46.358 p < 0.001
	Normal weight	46.6	37.7	22.0	19.8	
	Overweight	48.5	52.9	65.1	52.8	
	Obesity class I	3.9	8.7	12.8	22.6	
	Obesity class II	1.0	0.7	0.0	4.7	
WC	Very low	14.6	6.5	2.8	0.0	X^2^(9) = 81.059 p < 0.001
	Low	80.6	87.0	83.5	61.3	
	High	4.9	6.5	13.8	36.8	
	Very high	0.0	0.0	0.0	1.9	
CI	Normal	93.2	85.5	61.5	30.2	X^2^(3) = 122.524 p < 0.001
	Increased	6.8	14.5	38.5	69.8	
MA status	≤ CA	62.1	66.7	54.1	50.9	X^2^(3) = 7.685 p = 0.053
	> CA	37.9	33.3	45.9	49.1	
TBW	Below healthy	2.9	5.1	5.5	19.8	X^2^(6) = 27.151 p < 0.001
	Healthy	95.1	92.0	93.6	78.3	
	Above healthy	1.9	2.9	0.9	1.9	
%FM	Underweight	1.9	1.4	1.8	2.8	X^2^(9) = 79.199 p < 0.001
	Healthy	73.8	62.3	43.1	22.6	
	Overweight	15.5	24.6	45.9	43.4	
	Obese	8.7	11.6	9.2	31.1	
PA readiness	Able to perform PA	88.3	76.8	69.7	34.0	X^2^(6) = 83.881 p < 0.001
	Able to perform PA with restrictions	5.8	18.1	20.2	40.6	
	Unable to perform PA	5.8	5.1	10.1	25.5	

BMI = body mass index; CA = chronological age; CI = conicity index; MA = metabolic age; PA = physical activity; PO = police officer; TBW = total body water; WC = waist circumference; %FM = fat mass percentage.

## DISCUSSION

This study aimed to characterize the morphological profile and PA readiness of Portuguese POs according to sex and age group. Overall, the findings indicate a generally acceptable morphological profile. However, the high prevalence of excess Wt, increased %FM, and limited PA readiness observed in a substantial proportion of the sample highlights the need for continuous monitoring and targeted interventions focused on PF and body composition within police organizations.

Lifestyle behaviors are closely associated with body composition [^1^] and overall health. Consequently, adopting an active and healthy lifestyle remains one of the most effective strategies for preventing obesity and related health conditions. Previous studies have demonstrated that leisure-time PA is positively associated with body composition among POs, with physically active individuals exhibiting lower %FM and greater MM [^7^]. These findings reinforce the importance of promoting regular PA throughout police careers.

A favorable morphological profile and high levels of PF have consistently been associated with better health outcomes and enhanced occupational performance among POs [^13^]. Regular PA and balanced dietary habits play a key role in maintaining an adequate body composition, which is essential for meeting the physical and psychological demands of police work. Previous evidence suggests that POs with higher levels of PF are better prepared to respond to emergency situations, perform suspect apprehensions, and cope with other occupational demands, while also experiencing lower injury rates [^7^].

Furthermore, healthy lifestyle habits among POs may contribute not only to individual well-being but also to public safety. Physically fit POs tend to demonstrate greater alertness, faster response times, and improved capacity to manage high-stress situations, thereby enhancing the effectiveness of police services and contributing to community safety [^12^]. Promoting a culture of health and fitness within police organizations may therefore strengthen workforce resilience and operational readiness.

In the present study, the anthropometric profile of Portuguese POs indicated that 38.5% had a normal BMI, whereas 49.6% were classified as overweight and 11.6% as obese. Regarding central adiposity indicators, 78.2% presented low WC values, while 14.7% were classified as having high or very high WC. Furthermore, 67.8% of participants had a normal CI, whereas 32.3% presented an increased CI. Bioelectrical impedance analysis showed that 59.9% of POs had a MA lower than or equal to their CA, while 40.1% had a MA greater than their CA. Most participants presented healthy TBW levels (89.6%), whereas 8.4% and 2.0% were classified as below and above the healthy range, respectively. %FM was classified as healthy in 52.9% of participants, overweight in 30.0%, and obese in 13.7%. Finally, 65.8% of POs were classified as ready to perform PA, whereas 21.2% and 13.0% were classified as able to perform PA with restrictions and unable to perform PA without prior medical evaluation, respectively. This latter condition was more frequent among women than men (22.2% vs. 11.2%).

These findings, together with the decline in PA readiness observed across age groups (Tables 5 and 7), highlight the importance of implementing physical training programs throughout police careers to mitigate the adverse effects associated with the profession. Previous evidence indicates that the levels of PA during young adulthood are predictive of PF in middle age, after which occupational performance tends to decline more markedly [^2^]. Furthermore, PF decreases progressively with aging, with a more pronounced decline after the age of 50, estimated at approximately 1% per year [^11^].

In addition, body composition indicators and PA readiness differed according to sex (Table 3). When compared with POs from different countries and continents [^8^], Portuguese POs presented lower mean BMI (26.1 ± 3.2 kg/m^2^ vs. 28.0 ± 4.5 kg/m^2^) and %FM (22.4% ± 6.9% vs. 24.9% ± 6.4%). Nevertheless, excess Wt remains a relevant concern in this population. The high prevalence of overweight and obesity reported among POs worldwide has been attributed to multiple occupational, behavioral, and environmental factors, including irregular work schedules, poor dietary habits, insufficient PA, and the inherent unpredictability of police work [^8^].

Shift work, a characteristic feature of police occupations, has been associated with increased adiposity and reductions in MM [^9^]. Additional factors such as technological advances, vehicle-based policing, presenteeism, and years of service may also negatively affect body composition and contribute to the increased prevalence of obesity among POs [^1^,^8^]. These mechanisms may help explain the proportion of POs in the present study who exceeded recommended BMI and %FM thresholds.

Body composition differs between men and women, and POs are no exception. In the present study, female POs exhibited significantly lower values for Ht, Wt, BMI, HC, WC, WHR, WHtR, CI, the difference between MA and CA, TBW, absolute BM, and absolute MM than male POs. Conversely, female POs were significantly older and presented higher %FM values than their male counterparts.

When compared with female POs from the Buffalo Police Department, New York [^24^] (mean age: 44.0 ± 5.7 years; Ht: 166.3 ± 5.9 cm; Wt: 73.8 ± 14.6 kg; BMI: 26.7 ± 5.0 kg/m^2^; total %FM: 31.5% ± 6.4%; and total MM: 49.1 ± 6.5 kg), Portuguese female POs presented lower mean age (42.7 ± 11.8 years) and lower mean values for all morphological characteristics, including Ht (165.6 ± 4.3 cm), Wt (64.9 ± 7.3 kg), BMI (23.7 ± 2.8 kg/m^2^), total %FM (30.3% ± 6.3%), and total MM (42.7 ± 3.4 kg).

Similarly, compared with female POs from a northeastern city in the U.S. [^24^], who presented a mean age of 41.5 ± 6.1 years, BMI of 26.1 ± 4.8 kg/m^2^, WC of 80.3 ± 12.0 cm, and total %FM of 30.9% ± 6.0%, Portuguese female POs exhibited a higher mean age (42.7 ± 11.8 years) and lower mean BMI (23.7 ± 2.8 kg/m^2^), WC (78.8 ± 8.8 cm), and total %FM (30.3% ± 6.3%).

Among female POs, age group had a significant effect on Ht, BMI, WC, WHR, WHtR, CI, MA, and %FM. Specifically, POs aged ≤ 29 years presented higher TBW values, whereas those aged 30-39 years exhibited higher Ht values. In contrast, POs aged ≥ 50 years consistently presented higher values for the remaining morphological attributes. These findings are consistent with the well-established influence of sex and age on body composition since both fat-free mass (FFM) and %FM vary according to these factors. Because FFM contains a substantially higher proportion of TBW than %FM, younger and male POs, who generally exhibit greater FFM and lower %FM, tend to present higher hydration levels. This physiological relationship may explain the differences in body composition observed across age groups and between sexes [^14^]. In particular, it helps explain why POs aged ≤ 29 years exhibited higher TBW values, whereas those aged ≥ 50 years presented higher values for most of the remaining morphological attributes.

Among male POs, age group had a significant effect on Ht, Wt, BMI, WC, WHR, WHtR, CI, MA, TBW, absolute BM, %FM, and total MM. Specifically, POs aged ≤ 29 years exhibited higher TBW values, whereas those aged 30-39 years presented higher Ht, BM, and absolute MM values. In contrast, POs aged ≥ 50 years consistently presented higher values for the remaining morphological attributes.

When compared with male POs from the Buffalo Police Department, New York [^25^] (mean age: 44.0 ± 8.7 years; Ht: 178.8 ± 9.5 cm; Wt: 94.5 ± 14.2 kg; BMI: 29.8 ± 5.8 kg/m^2^; total %FM: 23.4% ± 5.4%; and total MM: 69.9 ± 6.7 kg), Portuguese male POs presented lower mean age (39.7 ± 11.0 years) and lower mean BMI (26.5 ± 3.1 kg/m^2^) and total %FM (20.8% ± 5.9%). Similarly, compared with male POs from a city in the northeastern U.S. [^26^] (mean age: 43.4 ± 8.4 years; BMI: 30.2 ± 3.7 kg/m^2^; WC: 99.3 ± 10.5 cm; and total %FM: 24.0% ± 5.1%), Portuguese male POs exhibited lower mean age (39.7 ± 11.0 years), BMI (26.5 ± 3.1 kg/m^2^), WC (91.7 ± 8.5 cm), and total %FM (20.8% ± 5.9%).

Older age is generally associated with longer service duration in police work. According to the systematic review conducted by Kukić et al. [^1^], years of service may negatively influence body composition by increasing obesity prevalence and cardiovascular disease risk among POs. Taken together, the present findings support the notion that body composition and PA readiness among POs are influenced not only by sex but also by age group.

From an occupational perspective, these differences highlight the importance of monitoring body composition throughout the police career, as reductions in FFM and increases in %FM, particularly with advancing age, may negatively affect physical performance, metabolic health, and readiness for duty. Consequently, implementing strategies aimed at preserving lean mass and adequate hydration while preventing excessive fat accumulation is essential for maintaining operational fitness and long-term health among POs.

POs are at increased risk of obesity due to the combination of prolonged sedentary periods associated with administrative duties and the occupational demands of operational activities. Therefore, evidence-based strategies are needed to prevent and manage obesity and other related chronic health conditions. Furthermore, because the levels of PA tend to decline with age, police organizations should promote working environments and institutional policies that support the maintenance of PF throughout an officer’s career [^10^].

Demling & DeSanti [^9^] recommend combining a hypocaloric, high-protein diet with resistance training to improve body composition and overall health among POs. This approach may reduce %FM while preserving MM, which is essential for maintaining physical performance and long-term health. Similarly, Raju et al. [^25^] advocate the implementation of comprehensive programs promoting healthy eating habits and regular PA. Such interventions may help prevent obesity, reduce the risk of metabolic complications, and improve overall well-being among POs.

Findings from other police organizations, including the London Metropolitan Police [^27^], the Florida State University Police Department [^28^], the St. Petersburg Police Department [^29^], the Albany Police Department [^30^], and the Edmonton Police Service [^31^], further suggest that the implementation of periodic PF assessments may represent one of the most effective strategies for monitoring health status, maintaining operational readiness, and promoting long-term occupational health among POs.

Despite the initial steps taken toward establishing evidence-based strategies to improve POs’ body composition, the consistent implementation of such initiatives in routine practice remains limited in most countries. Future interventions should be tailored to account for differences related to sex and age, ensuring that they are both inclusive and comprehensive. The adoption of a nationwide strategy may help support the PF of POs across different territorial commands of the PSP, thereby contributing to improved health outcomes and operational effectiveness.

In the long term, sustained investment in structured PF and health monitoring programs may not only improve officers’ well-being but also strengthen the operational readiness and resilience of the police force as a whole, ultimately contributing to enhanced public safety and service quality.

## CONCLUSIONS

Regarding the morphological profile of Portuguese POs, 11.6% were classified as having obesity class I or II according to BMI, 14.7% presented high or very high WC values, 32.2% exhibited an increased CI, 40.1% had a MA greater than or equal to their CA, 8.4% presented TBW values below the healthy range, and 43.7% were classified as overweight or obese according to %FM. In addition, 21.2% of participants were classified as able to perform PA with restrictions, whereas 13.0% were classified as unable to perform PA without prior medical evaluation.

These findings highlight the need for institutional strategies that promote active and healthy lifestyles and support the maintenance of PF throughout the police career. Efforts to improve POs’ health and quality of life should begin with occupational health services capable of identifying health risks at an early stage and providing timely intervention. In this context, the establishment of dedicated occupational health structures within the PSP, together with an Applied Sport Sciences unit incorporating areas such as exercise prescription and nutritional guidance, may facilitate the implementation of a systematic framework for assessing and monitoring the biomotor and morphological profiles of POs.

## Data Availability

Upon publication, the data will be made available by the authors upon request. The data are not publicly available due to ethical restrictions.
